# Unusual microscopic changes of Ameloblastic Fibroma and Ameloblastic Fibro-odontoma: A systematic review

**DOI:** 10.4317/jced.55460

**Published:** 2019-05-01

**Authors:** Saede Atarbashi-Moghadam, Mojtaba Ghomayshi, Soran Sijanivandi

**Affiliations:** 1 Associated professor, Department of Oral and Maxillofacial Pathology, School of dentistry, Shahid Beheshti University of Medical Sciences, Tehran, IR Iran; 2 Student, Dental Research Center, Research Institute of Dental Sciences, Shahid Beheshti University of Medical Sciences, Tehran, Iran

## Abstract

**Background:**

Ameloblastic fibroma (AF) and ameloblastic fibro-odontoma (AFO) are uncommon benign mixed odontogenic neoplasms. Although unusual microscopic changes including hybrid tumors have been documented in publications, their clinical outcome prediction and treatment modality selection are still challenging due to scarcity. Objective: Analysis of AF/AFO’s unusual microscopic variants in order to improve histopathologic diagnosis and to help clinicians in making informed treatment choices.

**Material and Methods:**

An electronic search was performed in PubMed’s database using keywords: “ameloblastic fibroma”, “ameloblastic fibroodontoma”, “ameloblastic fibro-odontoma”. The search scheme was limited to articles in English, dated ‘January 1998’ to ‘October 2018’, with full texts (case reports and series) and human studies. Eligibility criteria included publications having enough clinical, radiological, and histological data to confirm their diagnosis. Age, sex, lesions’ location, radiologic features, signs, symptoms, treatment approaches, and recurrences were recorded and analyzed.

**Results:**

In this systematic review, 11 articles (reporting 14 cases) were selected. Patients’ mean age was 13.75 years (male/female = 1.8). The posterior region of the mandible was the lesions’ commonest location (57.14%). Swelling was reported in 78.57% of the cases, pain in 28.57% but 21.42% were asymptomatic. Radiolucent unilocular appearance was the commonest radiographic feature, but 28.57% of the cases showed a mixed radiolucent-radiopaque appearance. Other reported radiographic findings were impacted tooth (78.57%), root resorption (28.57%), tooth mobility (35.71%), and cortical perforation (14.28%). No recurrences were reported. Calcifying odontogenic cyst (COC) was the commonest lesion associated with AF/AFO (53.33%). Unicystic ameloblastoma and cystic changes without prominent epithelial lining were other reported hybrid lesions. Reported microscopic variations were pigmentation and ghost cell differentiation.

**Conclusions:**

COC was the commonest lesion associated with AF/AFO. Although COC commonly occurs in the jaws’ anterior region, hybrid cases were more common in the posterior area. No malignant transformations were reported. The treatment modality is mostly chosen based on the lesion’s most aggressive part.

** Key words:**Ameloblastic fibroma, Ameloblastic fibro-odontoma, Odontogenic tumor, Jaw.

## Introduction

Ameloblastic fibroma (AF) is an uncommon benign odontogenic neoplasm, which is described by the proliferation of both the odontogenic epithelium and the mesenchyme. Ameloblastic fibro-odontoma (AFO) is demarcated as a lesion with the microscopic structures of an AF that also contains dental structures, namely dentine and enamel (1,2). Some researchers have designated that when only dentin matrix and dentinoid material is produced, the lesion should be called ameloblastic fibro-dentinoma (AFD) (2). AFO and AFD are not currently considered as separate entities as recently suggested in the 4th edition of WHO classification and they are currently supposed as part of the spectrum of microscopic changes seen in a developing odontoma. However, it is recognized that AFO and AFD can reach large sizes and they can arise in age groups inconsistent with a hamartoma. Moreover, it has been suggested that these lesions could have some features that are not supportive of the concept that they will progress into odontomas. It has also been suggested that some AFOs and AFDs may be true neoplasms (3).

These lesions are mostly diagnosed in the first two decades of life with a slight male predilection. The posterior region of the mandible is reported as their most common location. Large neoplasms exhibit a painless, slow-growing swelling, which may lead to delayed eruption, tooth mobility or tooth displacement. Buchner *et al.* (2) proposed that there are two different types of AFs: one of neoplastic nature and the other representing a hamartomatous lesion. Radiographically, AF shows either a unilocular or a multilocular radiolucency, both with well-defined borders. AFO contains a variable amount of calcified material with the radiodensity of tooth structures (1,2,4). The treatment of choice for these lesions is a conservative excision. Recurrence is uncommon and they might have a potential for malignant transformation (4,5). Rarely, AF/AFO are associated with other odontogenic cysts and tumors or show rare microscopic changes (1,4,6-15). The aim of this systematic review is gathering data about unusual variations of these tumors and discussing their clinical, radiographic and histopathologic features.

## Material and Methods

An electronic search was performed in PubMed’s database using the following keywords: “ameloblastic fibroma” (107 references), “ameloblastic fibroodontoma”, and “ameloblastic fibro-odontoma” (454 references). The search scheme was limited to articles in the English language, published between ‘January 1998’ and ‘October 2018’, with full texts (case reports and case series) and human studies. Initially, titles and abstracts of the articles were studied then unrelated articles were omitted. References of the selected published reports were also searched manually.

**Figure 1:**
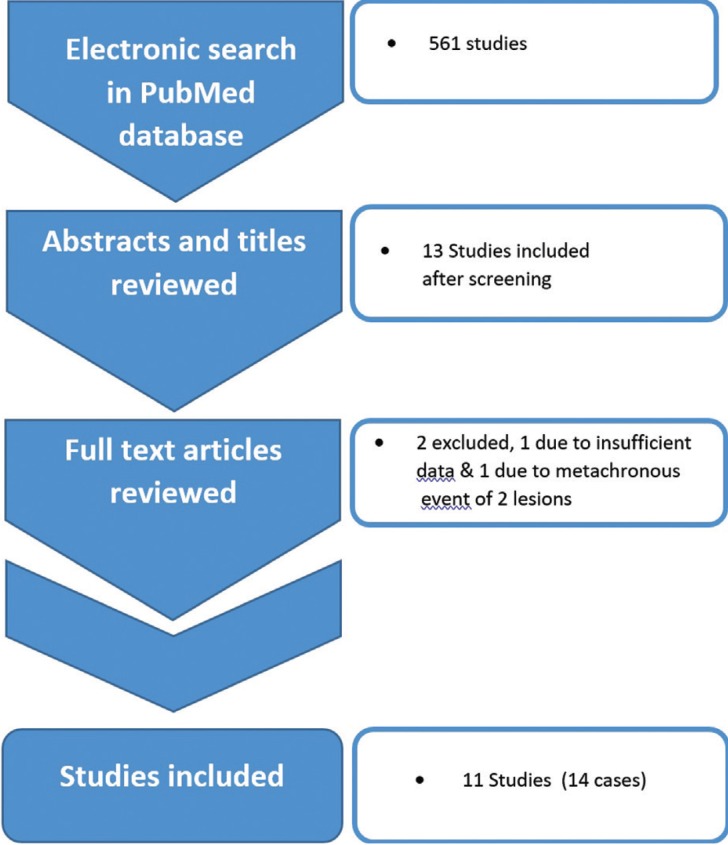
literature search’s strategy diagram.

Articles with sufficient clinical, radiologic, and microscopic data, which confirmed the diagnosis of AFs or AFOs with unusual microscopic findings, were selected (Fig. 1). Material achieved from all the cases were assessed in detail and AF/AFO associated with other cysts & neoplasms or showing rare histopathologic changes were extracted. Finally, the clinical and radiographic information of cases reported in the selected articles were evaluated including patients’ age and sex, lesions’ location, signs, symptoms, recurrences, and radiologic features such as content, loculation, tooth impaction, tooth displacement, and root resorption.

## Results

**Figure 2:**
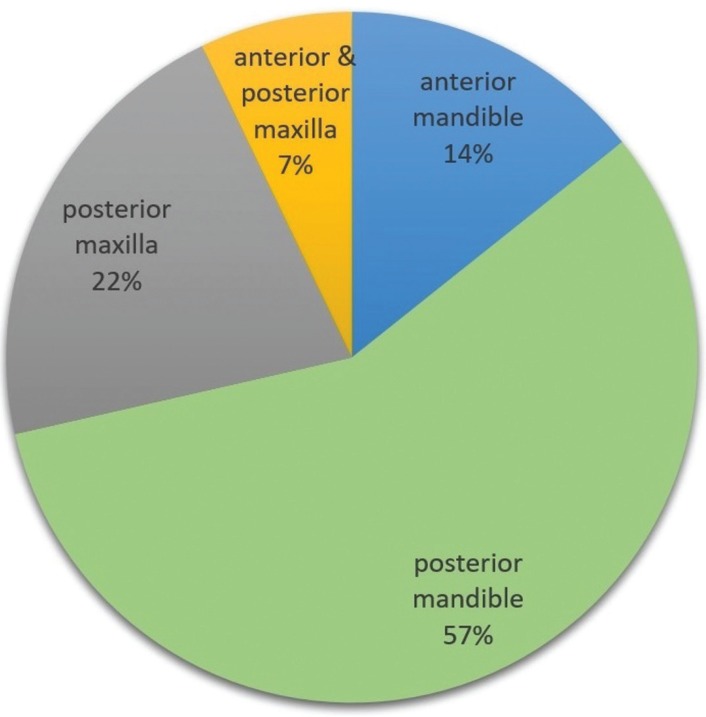
Lesion Locations’ Comparison Diagram.

In this systematic review, 11 articles were selected in which 14 cases were reported. A data summary of the cases is displayed in  Table 1. Patients’ ages ranged from 3.5 to 26 years (mean 13.75). There were 9 (64.28%) male and 5 (35.71%) female patients with a male to female ratio of 1.8:1. The mandible was the most frequently involved site (71.42%) and the posterior region was the commonest location (57.14%) of the lesions (Fig. 2). The majority of patients showed swelling (78.57%), pain was documented in 28.57% of the cases and 21.42% were asymptomatic.

**Table 1:**
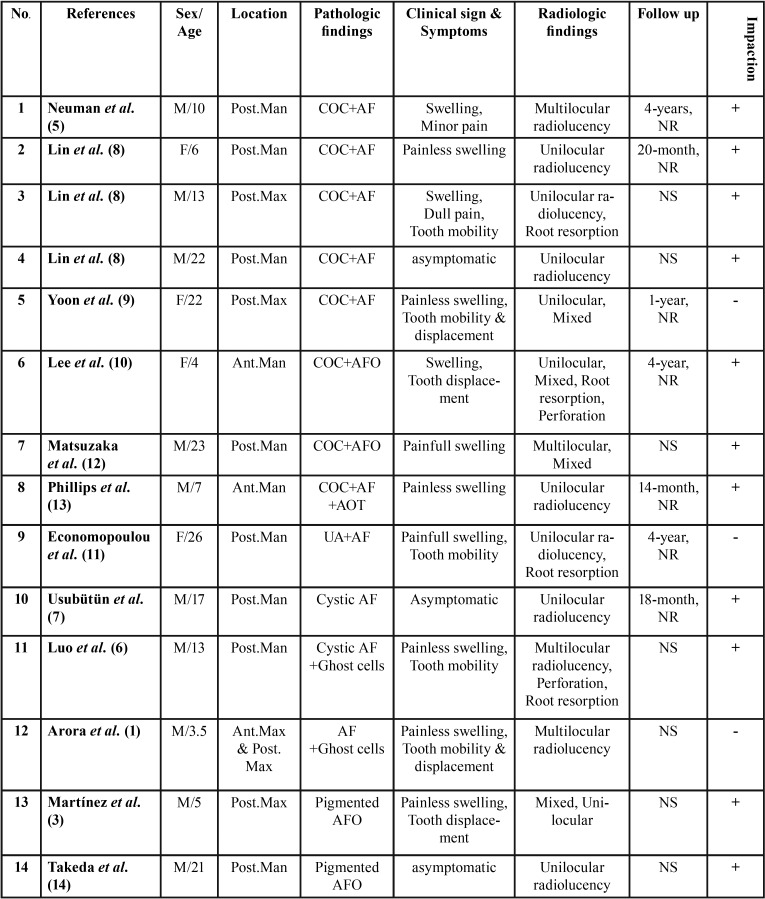
AF/AFO associated with other lesions or unusual histopathology.

Radiographically, 71.42% of the lesions were unilocular. Only 28.57% showed a mixed radiolucent-radiopaque appearance, while about 78.57 % of the cases contained impacted teeth. Root resorption and tooth mobility were reported in 28.57% (4 cases) and 35.71% (5 cases), respectively. Cortical perforation was seen in 2 cases (14.28%). The lesions’ sizes ranged from 1.5 to 6 centimeters. One case (reference 12) reported a history of blunt trauma one year prior to the diagnosis.

Histopathologically, the lesion most commonly associated with AF/AFO was calcifying odontogenic cyst (COC) (53.33%). One case of unicystic ameloblastoma (UA) with AF was reported. Two cases of cystic AF without prominent epithelial lining have also been documented. Pigmentation (2 cases) and ghost cell differentiation (2 cases) were unusual microscopic findings.

## Discussion

In the present review, the lesions occurred almost twice as frequently in males as in females. Typical AF is slightly more common in males (2,4). AF/AFO tend to occur in the first two decades of life and in the posterior region of the mandible (1,2,4). The mean age of patients with rare variants of AF/AFO in our study was 13.75 years and the commonest location was the posterior region of the mandible, which is similar to the conventional type. Small AFs are asymptomatic and large neoplasms show a painless, slow-growing swelling (4). In the current review, the majority of cases showed swelling. Pain was reported in 28.57% of the patients. However, 21.42% of them were asymptomatic. Common AF/AFO rarely show root resorption and cortical perforation (4). Furthermore, the current study shows that tooth mobility, tooth displacement, root resorption and cortical perforation may occur but are uncommon. Most of the conventional AF/AFOs are associated with impacted teeth and unilocular radiolucencies (2,4), which is almost identical to the results found in this study. In this systematic review, some hybrid tumors showed huge sizes and destructive lesions (1,7,9), which support the tumoral nature of these lesions. It has been suggested by a number of researchers that hybrid tumors are not two separate lesions but are related to the multi-potential nature of the odontogenic epithelium (10,16). On the other hand, Neuman *et al.* (6) claimed that in some cases there are two distinct entities arising in the same location.

The lesion most commonly associated with AF/AFO was COC. Although COC tends to occur in the anterior region of the jaws, the hybrid cases were more common in the posterior area (75%). Some authors mentioned that the epithelial lining of COC shows the ability to induce the formation of dental structures in the underlying stroma. Thus, some odontogenic neoplasms may arise from this cyst (11). On the other hand, Altini and Farman (17) proposed that the development of the COC part is a secondary occurrence within the pre-existing odontogenic neoplasm. In addition, ghost cell changes have been reported without the COC component (1,7). The ghost cells in AF/AFO, have been reported only in the cystic component and/or the epithelial islands. Ghost cell changes have also been reported in odontoma, ameloblastoma, adenomatoid odontogenic tumor, craniopharyngiomas and pilomatricomas (7,18,19). Abnormal keratinization is the commonest proposed nature of ghost cells (18). Dentinogenic ghost cell tumors (DGCT) show ameloblastic epithelial islands containing ghost cells and dentinoid material. The presence of dental papilla-like connective tissue leads to the diagnosis of AF/AFO with ghost cell changes from DGCT that has a fibrous stroma (20). Apart from COC, other cystic changes have also been reported. Cystic formation in AF/AFO is a rare event, even in the epithelial component (8). Some authors proposed that damage, degeneration, and hemorrhage in the tissue may cause a cavitation that is secondarily epithelialized (21). Usubütün et al. (8) documented a cystic AF with no epithelial lining. In addition, Luo *et al.* (7) reported a cystic AF partially lined by a single layer of flattening epithelial cells. A metachronous AFO and dentigerous cyst have been reported, but they were not considered as one hybrid tumor because they were not synchronous events (22).

In our review, only one case was reported to have a history of trauma prior to the hybrid UA+AF (12). Chen *et al.* (23) proposed that if hard tissue components are not produced, and the collagenous stroma is replaced by the stromal mesenchymal tissue, AF could transform into an ameloblastoma.

Pigmented AF/AFO have also been reported (4,15). Melanocytes and melanin pigmentations are present in the skin, the nervous system, and the oral mucosa but do not typically exist within the bone (15). Dendritic cells containing melanin in these lesions may originate from the oral epithelium and the dental lamina or the neural crest (4). COC is the type of pigmented odontogenic lesion most frequently reported, followed by the odontogenic keratocyst. Most of the pigmented odontogenic lesions were reported in Blacks and Asians (24). Thus, racial pigmentation may be an important predisposing factor (15).

Finally, hyaline bodies of Rushton have also been reported in AF. They have been found in the cell-rich mesenchyme adjacent to the ameloblastic islands (25). These structures were found in odontogenic cysts and were rarely reported in odontogenic tumors like ameloblastoma (26). They are specific, non-keratin products of the odontogenic epithelium that are restricted to the epithelial lining and are rarely seen in the isolated areas of the subepithelial connective tissue (25).

The rarity of hybrid odontogenic lesions prevents an acceptable data report regarding treatment modalities; nevertheless, the treatment has been usually based on the lesional part with the most aggressive behavior (6). The treatment modality for most of these hybrid lesions has been conservative surgery (1,7-11,14) except one case in which “marginal resection” was carried out for the patient, because of the combination of UA and AF (12). Lin *et al.* (9) suggested that although simple excision appears to be enough for these hybrid lesions, long term follow-ups and additional cases are required to explain their biologic behavior (9). In this review, six cases had been followed up and no recurrences were documented (8-12,14). According to previous case reports, it seems that pigmentation and the presence of ghost cells do not affect the prognosis of AF/AFO (1,5,8,16).

In conclusion, hybrid types and AF/AFO with rare microscopic features are more common in lower ages, males, and the posterior region of the mandible. They often show painless swelling. COC is the most commonly reported associated lesion. Malignant transformations or atypical cells have not been reported. Choice of treatment modality appears to be mostly based on the most aggressive part of the lesion. The oral pathologists’ familiarity with unusual histopathologic changes is a necessity for correct histologic diagnosis and treatment.
